# A new classification for heterotopic ossification following periacetabular osteotomy with and without concomitant hip arthroscopy

**DOI:** 10.1302/2633-1462.611.BJO-2025-0222.R1

**Published:** 2025-11-11

**Authors:** Ta-Wei Tai, Diego J. Restrepo, Sergio F. Guarin Perez, Adrian E. Gonzalez-Bravo, Onur Hapa, Rafael J. Sierra

**Affiliations:** 1 Department of Orthopedics, National Cheng Kung University Hospital, College of Medicine, National Cheng Kung University, Tainan, Taiwan; 2 Department of Orthopedic Surgery, Mayo Clinic, Rochester, USA; 3 Department of Orthopedic Surgery, Dokuz Eylül University, İzmir, Turkey

**Keywords:** Periacetabular osteotomy, Heterotopic ossification, Cumulative probability, Classification, heterotopic ossification (HO), prophylaxis, arthroscopic procedures, surgical excision, Radiographs, total hip arthroplasty (THA), concomitant hip arthroscopy, low-dose aspirin, ossification

## Abstract

**Aims:**

Heterotopic ossification (HO) is a recognized complication following periacetabular osteotomy (PAO), but its patterns, incidence, and clinical relevance remain poorly defined. The commonly used Brooker classification, developed for total hip arthroplasty, is not fully applicable to HO after PAO. This study aimed to propose a new classification system specific to HO after PAO, evaluate its clinical relevance, and report the cumulative probabilities of radiological and symptomatic HO.

**Methods:**

This retrospective study included 643 patients who underwent PAO between January 2006 and July 2024. A total of 308 patients (47.9%) had concomitant arthroscopic procedures. Radiographs were analyzed to identify HO using both the Brooker classification and a newly developed system. The cumulative probabilities of radiological and symptomatic HO were calculated using Kaplan-Meier analysis. Difference of variables between patients with and without HO was analyzed. Receiver operating characteristic (ROC) curve analysis was used to compare the predictive performance of the two classification systems.

**Results:**

The cumulative probabilities of radiological and symptomatic HO at five years post-PAO were 30.4% and 3.8%, respectively. The probabilities were 41.9% in males and 29.2% in females. Four patients (2.8%) required surgical excision of HO, all classified as type 3 or 4 under the new system. The ROC analysis demonstrated superior predictive performance for the new classification system (AUC = 0.928) compared to the Brooker classification (AUC = 0.697; p = 0.007). No significant differences were found between the patients with and without HO in terms of age, sex, concomitant arthroscopic procedures, or receiving prophylaxis in addition to low-dose aspirin.

**Conclusion:**

This study highlighted the cumulative probabilities of HO following PAO and introduced a new classification system that improved clinical relevance and predictive accuracy. These findings have provided insights into the incidence and management of HO after PAO, and laid the groundwork for standardized reporting and future research.

Cite this article: *Bone Jt Open* 2025;6(11):1436–1445.

## Introduction

Periacetabular osteotomy (PAO) is a surgical procedure designed to correct various acetabular deformities and redistribute the load through the hip joint.^1-3^ This intervention can alter the natural course of hip conditions and slow the progression of arthritis in patients with hip dysplasia, and is also used to treat other structural disorders of the hip such as acetabular retroversion and protrusio. In addition to PAO, arthroscopic techniques have become a widely used approach for hip preservation, particularly when intra-articular lesions are present. Recently, combining PAO with hip arthroscopy has gained popularity as an effective strategy to address both bony deformities and labral lesions simultaneously. This combined approach has demonstrated favourable outcomes, offering patients comprehensive treatment in a single surgical setting.^4^

Despite advancements in surgical techniques over recent decades, including the development of minimally invasive and muscle-sparing approaches, PAO remains a complex procedure associated with a significant risk of complications.^2,3,5^ Among these complications, heterotopic ossification (HO) remains relatively understudied. However, its occurrence can result in persistent hip pain and restricted range of motion, potentially necessitating reoperation to restore joint function and alleviate symptoms.

HO following total hip arthroplasty (THA) and hip arthroscopy has been extensively studied, with reported incidence rates of approximately 30% after THA and less than 5% following hip arthroscopy.^6-9^ However, the available literature on HO after PAO is limited to case reports and small series, with most studies focusing on outcomes other than HO as their primary endpoint.^10-15^ The primary endpoints of most of these studies were not HO. One case series of hip arthroscopy, with or without PAO, suggested that the incidence of HO could be reduced to 1.5% with prophylactic use of non-steroidal anti-inflammatory drugs (NSAIDs).^10^ A multicentre study on PAO complications reported that 34 of 205 patients (16.6%) developed HO, with only one case (0.5%) requiring surgical excision.^11^ Most studies have relied on Brooker’s classification system,^16,17^ developed for HO after THA, despite the distinct pattern of HO observed following PAO. To date, no classification system specific to HO after PAO has been established.

This study aims to address these gaps by reporting the cumulative incidence and types of HO after PAO in a large case series. Radiological and symptomatic HO, along with cases requiring excision, will be analyzed. Additionally, based on the observed patterns of HO following PAO, we propose a new classification system to better characterize and manage this condition.

## Methods

### Patient cohort and data collection

This study received approval from the institutional review board of the Mayo Clinic. Patients who underwent PAOs performed by a single surgeon (RJS) specializing in young hip conditions between January 2006 and July 2024 were identified. Indications for surgery included hip dysplasia, acetabular retroversion, and protrusion. Patients without documented radiological follow-up of at least two months post-surgery were excluded. Postoperative radiographs, ranging from the immediate postoperative period to the final follow-up, were evaluated for the presence and classification of HO. Electronic medical records were reviewed to collect demographic data, medical and social history, previous hip surgeries, indications for PAO, and any concomitant arthroscopic procedures.

A total of 728 patients were enrolled for initial screening. We excluded 18 patients who underwent concomitant femoral derotational osteotomy, 17 who had surgical hip dislocation, and 50 who had no available radiographs or only imaging within the first two months after surgery. Ultimately, 643 patients were included in the study, with a mean age of 24.7 years (12 to 48, SD 8.5) (Table I). The cohort comprised 565 females and 78 males, with a mean BMI of 25.3 kg/m² (15.4 to 50.9, SD 4.8). There were 544 cases of hip dysplasia (84.6%), 69 acetabular retroversion (10.7%), 29 combined dysplasia and retroversion (4.5%), and one case of protrusio (0.2%). In total, 308 patients (47.9%) underwent a concomitant arthroscopic procedure; 222 (34.5%) had labral repair, 144 (22.4%) had cam osteochondroplasty, and 194 (30.2%) had acetabular chondroplasty. The median radiological follow-up in our cohort was 1.5 years (IQR 0.6 to 3.0).

**Table I.** Basic characteristics of patients who presented with or without heterotopic ossification (HO) following periacetabular osteotomy.

Characteristic	Total (n = 643)	With HO (n = 143)	Without HO (n = 500)	p-value
**Mean age, yrs (SD)**	24.7 (8.5)	24.2 (8.5)	24.9 (8.5)	0.445*
≥ 20 yrs, n (%)	407	87 (21.4)	320 (78.6)	0.489†
< 20 yrs, n (%)	236	56 (23.7)	180 (76.3)	
**Sex, n (%)**				
Female	565	121 (21.4)	444 (78.6)	0.177†
Male	78	22 (28.2)	56 (71.8)	
**Mean BMI, kg/m² (SD)**	25.3 (4.8)	25.7 (5.0)	25.1 (4.7)	0.219*
**Medical history, n (%)**				
Hypothyroidism	28	6 (21.4)	22 (78.6)	0.916†
Asthma	76	22 (28.9)	54 (71.1)	0.134†
Gastroesophageal reflux disease	30	7 (23.3)	23 (76.7)	0.883†
Diabetes	8	2 (25.0)	6 (75.0)	0.850†
Anaemia	19	4 (21.1)	15 (78.9)	0.900†
**Social history, n (%)**				
Smoking	90	14 (15.5)	76 (84.4)	0.102†
Substance abuse	5	1 (20.0)	4 (80.0)	0.090†
Previous hip surgery	102	24 (23.5)	78 (76.5)	0.733†
**Indication for PAO, n (%)**				
Isolated dysplasia	544	117 (21.5)	427 (78.5)	0.295†
Isolated acetabular retroversion	69	19 (27.5)	50 (72.5)	0.263†
Dysplasia + retroversion	29	7 (24.1)	22 (75.9)	0.801†
Protrusio acetabuli	1	0 (0)	1 (100.0)	0.593†
**Arthroscopic procedure, n (%)**	308	65 (21.1)	243 (78.9)	0.507†
Labral repair	222	48 (21.6)	174 (78.4)	0.784†
Head/neck junction osteochondroplasty	144	28 (19.4)	116 (80.6)	0.360†
Acetabular chondroplasty	194	42 (21.6)	152 (78.4)	0.813†
**HO prophylaxis, n (%)** ‡	102	21 (20.6)	81 (79.4)	0.660†

A p-value < 0.05 indicates a statistically significant difference between the groups with and without HO.

* Independent-samples *t*-test.

† Chi-squared test.

‡ HO prophylaxis included the use of indomethacin or meloxicam.

PAO, periacetabular osteotomy.

### Procedures for PAO

The procedures were carried out as previously described.^18,19^ Patients were positioned supine on a radiolucent table to facilitate fluoroscopic guidance throughout the procedure. Preoperative baseline measurements of hip range of motion were documented. A longitudinal incision was made over the anterior superior iliac spine (ASIS) using a modified Smith-Petersen approach, with careful preservation of the abductor muscles and rectus femoris. To minimize soft-tissue injury, the sartorius muscle was meticulously detached from the ASIS using a scalpel. Retractors were strategically placed to protect vital structures, including the sciatic notch and surrounding areas of the pubic bone. During exposure and osteotomy of the ischium and pubis, the hip and knee were maintained in a flexed position. Full mobilization of the acetabular fragment was confirmed using a Schanz pin and a sharp bone hook before repositioning. Fluoroscopic evaluation of radiological landmarks, such as a flat sourcil and appropriate acetabular version, was performed to achieve optimal fragment placement.

Postoperatively, patients were instructed to follow a restricted weightbearing protocol, limited to toe-touch for the initial four weeks, with gradual progression as tolerated. Physical therapy was initiated three weeks after surgery to facilitate rehabilitation. Smoking cessation was strongly recommended to enhance bone healing and optimize surgical outcomes.

### Prophylaxis for heterotopic ossification

HO prophylaxis following PAO was primarily administered with aspirin 81 mg twice daily, which was also routinely used for six weeks to prevent thromboembolic events. An additional, short-term NSAID for HO prophylaxis was prescribed to 51% (40/78) of male patients and 11% (62/565) of female patients. The most commonly used regimens were indometacin 75 mg or meloxicam 15 mg per day for five days. The addition of a NSAID in males was incorporated as the surgeon perceived that the risk of HO was higher in muscular males.

### Classification for heterotopic ossification

The most commonly used classification system for HO around the hip is the Brooker classification, originally developed to categorize HO after THA.^16,17^ However, this system is not fully applicable to HO following PAO. The Brooker system includes exophytes from both the lateral pelvis and the distal femur, the latter being rarely observed after PAOs.

**Fig. 1:**
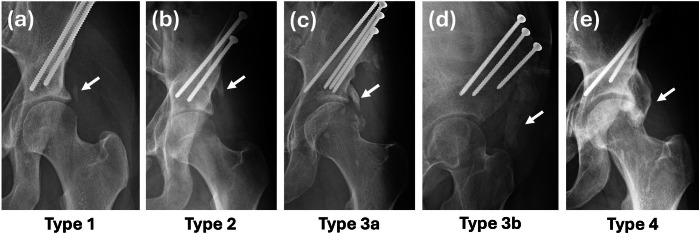
New classification system for heterotopic ossification following periacetabular osteotomy. a) Type 1 represents a small bony island, while b) Type 2 consists of a small bony spike originating from the osteotomy site. Type 3 is further divided into two subtypes: c) Type 3a is characterized by a large bony spike extending downward and laterally toward the sourcil area, while d) Type 3b involves a similar large bony spike extending downward but positioned anterior to the sourcil area, visible on a false-profile lateral radiograph. e) Type 4 denotes diffuse heterotopic bone formation around the joint line.

To address this gap, we developed a new classification system specifically tailored for HO following PAOs, based on radiological findings from our large cohort of patients (Table II and Figure 1). This system categorizes HO into four types: type 1, isolated bony islands in the soft-tissue; type 2, small bony spikes; type 3, large bony spikes extending to the level of the sourcil; and type 4, diffuse heterotopic bone formation. Type 3 is subdivided into two subtypes based on the location of the bony spike: type 3a involves the lateral aspect of the hip joint, while type 3b involves the anterior aspect. Anterior spikes are typically best visualized on lateral radiographs, such as the false-profile view. The distinction between these subtypes is clinically significant because anterior bony spikes (type 3b) are more likely to interfere with hip flexion and may be associated with pain and limitations in range of motion. To assess interobserver reliability of the proposed classification system, a random sample of 50 radiographs was independently reviewed by three orthopaedic surgeons (TWT, DJR, SFG). Pairwise agreement was evaluated using Cohen’s weighted kappa with linear weights.

**Table II.** Classification of heterotopic ossification following periacetabular osteotomy.

Classification	Definition
Type 1	Bony islands in the soft-tissue around the hip joint
Type 2	Small spikes originating from the iliac osteotomy site
Type 3	Large spikes extending down to the sourcil level:
	**3a:** Lateral large spikes visible in the anteroposterior radiograph
	**3b:** Anterior large spikes visible in the false-profile lateral radiograph
Type 4	Diffuse heterotopic bone formation around the hip joint line

Receiver operating characteristic (ROC) curve analysis was performed to evaluate the ability of the Brooker classification and the new classification to predict symptomatic HO. Symptomatic HO was defined as heterotopic bone formation associated with localized pain or limitation in range of motion, diagnosed by the senior orthopaedic surgeon (RJS) through clinical assessment and imaging, after excluding other potential causes such as labral pathology, residual deformity, tendinopathy, or osteoarthritis. The area under the curve (AUC) was calculated for both classification systems to assess their discriminative performance.

### Statistical analysis

Descriptive statistics summarized patient demographic information. Differences in continuous variables, such as age and BMI, between patients with and without HO were analyzed using independent-samples *t*-test. Similarly, binary characteristics were compared using a chi-squared test. Fisher’s exact test was applied to evaluate the distribution of HO types, addressing low expected frequencies in certain categories to ensure robust analysis. ROC curve analysis was performed and the AUCs were compared using the DeLong test for correlated ROC curves, which provided a *z*-score, p-value, and 95% CI for the AUC difference. A p-value < 0.05 indicates statistical significance. All statistical analyses were conducted using BlueSky Statistics software (version 10.3.4, USA) and SPSS (version 29.0, IBM, USA). The cumulative probability of HO was estimated using Kaplan-Meier analysis with log-rank test to account for censored follow-up data, and the corresponding plot was generated using GraphPad Prism software (version 10.0, USA).

## Results

**Fig. 2:**
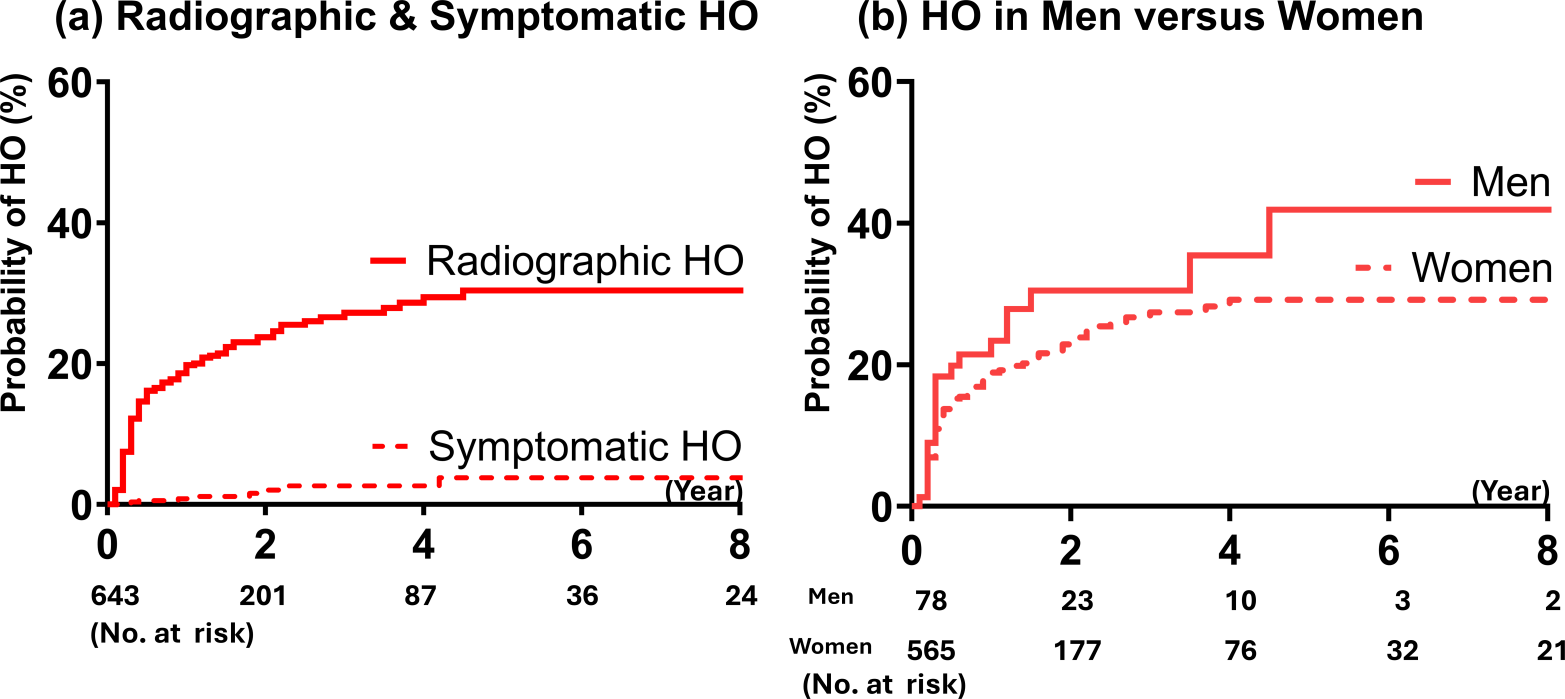
a) Kaplan-Meier analysis of the cumulative incidence of radiological and symptomatic heterotopic ossification (HO). The estimated probabilities of developing radiological and symptomatic HO at five years post-surgery are 30.4% and 3.8%, respectively. b) The estimated five-year probabilities of developing radiological HO in males and females are 41.9% and 29.2%, respectively; however, the difference was not statistically significant (p = 0.189, log-rank test).

Radiological analysis identified 143 patients with HO, and 500 without (Table I), yielding a crude overall incidence of 22.2%. No significant differences were found between the groups with and without HO in terms of age, sex, BMI, medical history, indications for PAO, various concomitant arthroscopic procedures, or receiving additional NSAID HO prophylaxis. Our analysis revealed no significant association between HO and any surgical scenario, including PAO alone, staged arthroscopy with PAO, concomitant arthroscopy with PAO, or previous arthroscopy combined with concomitant arthroscopy with PAO (p = 0.899) (Table III). Consequently, no specific risk factors for HO development could be identified. Considering censored follow-up data, the cumulative probabilities of radiological and symptomatic HO at five years post-surgery were 30.4% and 3.8%, respectively. Most cases of HO were observed within the first two years, with nearly all cases identified by five years postoperatively (Figure 2a). Males tended to have greater probabilities in developing HO than females (41.9% vs 29.2%), but the difference did not reach a significant difference (p = 0.189, log-rank test) (Figure 2b).

**Table III.** Incidence of heterotopic ossification (HO) in patients undergoing periacetabular osteotomy with or without arthroscopy.

Scenarios	With HO (n = 143)	Without HO (n = 500)	p-value*
PAO alone	58 (23.3)	191 (76.7)	0.899
Staged arthroscopy + PAO	20 (23.3)	66 (76.7)	
Concomitant arthroscopy with PAO	61 (20.9)	231 (79.1)	
Previous arthroscopy + concomitant arthroscopy with PAO	4 (25.0)	12 (75.0)	

* Chi-squared test.

PAO, periacetabular osteotomy.

Notably, all patients who developed symptomatic HO had not received additional prophylactic medication (Table IV). Among males, those who received additional prophylaxis had a lower incidence of HO compared to those who did not (25% vs 31.6%); however, the difference was not statistically significant (p = 0.614). Similarly, no significant difference was observed in the incidence of HO between females who received additional prophylaxis and those who did not (17.7% vs 21.9%, p = 0.515).

**Table IV.** Incidence of heterotopic ossification (HO) in males and females with and without prophylaxis.

Type of HO	Patients with HO prophylaxis, n (%)	Patients without HO prophylaxis, n (%)	p-value*
Male	40	38	
Radiological HO	10 (25)	12 (31.6)	0.617
Type 3 or 4 HO	3 (7.5)	3 (7.9)	> 0.999
Symptomatic HO	0	1 (2.6)	0.487
HO requiring excision	0	1 (2.6)	0.487
Female	62	503	
Radiological HO	11 (17.7)	110 (21.9)	0.515
Type 3 or 4 HO	0	14 (2.8)	0.384
Symptomatic HO	0	8 (1.6)	0.317
HO requiring excision	0	3 (0.6)	0.542

* Fisher’s exact test.

The distribution and clinical presentation of HO following 143 PAOs were assessed using the Brooker classification and a newly proposed classification system specific to PAO (Table V and Table VI). Based on the Brooker classification, 45 cases (31.5%) were classified as type 1, 95 cases (66.4%) as type 2, and three cases (2.1%) as type 3, with no type 4 cases identified. Symptomatic HO, defined as HO causing pain or restricted range of motion, was observed in eight patients (8.4%) with type 2 HO and in one patient (33.4%) with type 3 HO, showing a significant difference in the distribution among classifications (p = 0.024). HO requiring excision was reported in three patients with type 2 HO and one patient with type III HO, but this difference was not statistically significant (p = 0.055).

**Table V.** Presentation of heterotopic ossification following periacetabular osteotomy categorized by the Brooker classification system.

Heterotopic ossification	Brooker classification				p-value*
	Type 1	Type 2	Type 3	Type 4	
Radiological	45	95	3	0	
Symptomatic	0	8 (8.4%)	1 (33.4%)	0	0.024
Requiring excision	0	3 (3.2%)	1 (33.4%)	0	0.055

Radiological heterotopic ossification refers to cases found on radiographs regardless of symptoms. Symptomatic heterotopic ossification causes pain or limited range of motion. A p-value < 0.05 indicates a statistically significant difference.

* Fisher’s exact test.

**Table VI.** Presentation of heterotopic ossification following periacetabular osteotomy categorized by the proposed new classification system specific for periacetabular osteotomy.

Heterotopic ossification	Proposed classification					p-value*
	Type 1	Type 2	Type 3a	Type 3b	Type 4	
Radiological	45	78	10	3	7	
Symptomatic	0	1 (1.3%)	2 (20.0%)	2 (66.7%)	4 (57.1%)	< 0.001
Requiring excision	0	0	1 (10.0%)	2 (66.7%)	1 (14.3%)	< 0.001

Radiological heterotopic ossification refers to cases found on radiographs regardless of symptoms. Symptomatic heterotopic ossification causes pain or limited range of motion.

* Fisher’s exact test.

Using the proposed classification, interobserver reliability was substantial to excellent, with weighted kappa values of 0.718, 0.752, and 0.737 across the three pairwise rater comparisons (all p < 0.001; 95% CI 0.547 to 0.897). Overall, 45 cases (31.5%) were categorized as type 1, 78 cases (54.5%) as type 2, ten cases (7.0%) as type 3a, three cases (2.1%) as type 3b, and seven cases (4.9%) as type 4. Symptomatic HO was observed most frequently in type 3b and type 4, followed by type 3a and type 2, indicating a significant variation in distribution across classifications (p < 0.001, Fisher’s exact test). There were no significant differences in basic characteristics, medical history, or concomitant procedures between patients with mild HO (types 1 and 2) and those with higher grade HO (types 3 and 4) (Supplementary Material). HO requiring excision was also most prevalent in type 3b (2/3, 66.7%) and type 4 (1/7, 14.3%), followed by type 3a (1/10, 10.0%), with a statistically significant difference among classifications (p < 0.001, Fisher’s exact test). These findings highlight the clinical relevance of the new classification, particularly in identifying subtypes associated with greater symptom burden and the need for surgical intervention.

The ROC analysis demonstrated that the new classification system had a significantly higher discriminative ability than the Brooker classification in predicting symptomatic HO. The AUC for the Brooker classification was 0.697, indicating moderate predictive performance, whereas the AUC for the new classification was 0.928, reflecting excellent predictive accuracy. The DeLong test for comparing AUCs showed a statistically significant difference between the two classification systems (AUC difference = 0.231, z = -2.701, p = 0.007). The 95% CIs for the AUC difference ranged from -0.399 to -0.063, further confirming the superiority of the new classification system.

### Cases of excision and example

Four patients in our series required surgical excision of heterotopic bone due to persistent hip pain and limited range of motion. These included one case of type 3a, two cases of type 3b, and one case of type 4 HO according to the new classification. The cohort comprised three females and one male, with a mean age of 19.3 years (15 to 22; SD 3.1), which was significantly younger than other patients with HO (mean age 24.4 years (12 to 48; SD 8.6), p = 0.035). The mean interval between PAO and excision was 2.5 years (11.4 months to 4.6 years). Two patients underwent hardware removal, and one also received arthroscopic labral debridement for symptomatic labral fraying identified during preoperative evaluation. All patients who underwent HO excision experienced meaningful symptomatic improvement following surgery. One patient with type 3b received a single dose of 700 cGy and showed no recurrence during follow-up.

**Fig. 3:**
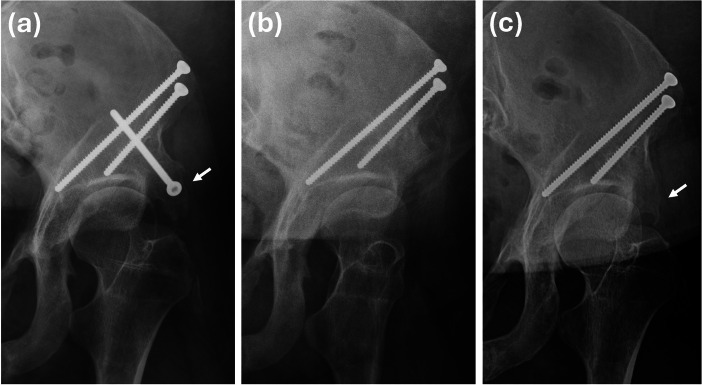
A case example of a 15-year-old female who underwent periacetabular osteotomy with retrograde screw fixation. a) Two years and four months post-surgery, she developed type 4 diffuse heterotopic bone formation. b) The retrograde screw and heterotopic bone were subsequently removed. Unfortunately, she experienced gradual recurrence of heterotopic bone formation after the excision. She underwent conservative treatment, and c) an anteroposterior radiograph taken four years after excision demonstrated a stationary condition of the heterotopic bone.

Recurrence was observed in a 15-year-old female who had initially undergone PAO with retrograde screw fixation for hip dysplasia. Her early postoperative recovery was uneventful, with satisfactory osteotomy healing. Two years after surgery, she experienced worsening groin pain and discomfort during hip flexion and internal rotation. Physical examination revealed tenderness over the anterosuperior iliac spine and retrograde screw head, and imaging confirmed type 4 diffuse HO (Figure 3). She underwent excision of the heterotopic bone and removal of the retrograde screw. Despite the intervention, gradual recurrence of HO was noted. Conservative management led to symptomatic improvement, and follow-up radiographs four years after excision revealed a stationary condition of the heterotopic bone, with the patient reporting only mild discomfort during internal and external rotation of the hip.

## Discussion

We aimed to report the cumulative probabilities of HO following PAO from a large cohort and proposed a new classification system to address this understudied issue. The cumulative probabilities for radiological and symptomatic HO were 30.4% and 3.8%, respectively. Notably, 2.8% of patients with HO required surgical excision, and all these cases were categorized as type 3 or 4 under the new classification system. These findings underscored the utility of the new classification in identifying clinically significant HO and facilitating targeted management strategies, enabling standardized comparisons for future research.

HO around the hip joint is a well-recognized complication following THA, hip arthroscopy, and PAO. The reported crude incidence of HO is approximately 30% after THA, while most studies report an incidence of less than 5% following hip arthroscopy.^6-9^ In the context of PAO, some studies have reported sporadic cases of heterotopic bone excision.^12-15^ A multicentre study on PAO complications from the Academic Network of Conservational Hip Outcomes Research (ANCHOR) group reported that 16.6% of patients (34 of 205) developed HO, distributed as 21 Brooker grade 1, seven grade 2, four grade 3, and two grade 4 cases.^11^ Among these, only one patient with grade 3 HO (0.5%) required surgical excision, while the remaining 33 cases were categorized as Clavien-Dindo grade I complications, requiring no medical treatment or intervention and not impacting the recovery process.^20,21^ Males have been reported to have a higher risk of developing HO compared to females.^7,9^ In our cohort, HO was observed in 21.4% of females (n = 121) and 28.2% of males (n = 22). The estimated five-year probabilities of developing radiological HO were also higher in males than in females. However, this difference did not reach statistical significance, which may be attributed to the higher proportion of males receiving additional prophylactic medication. These findings highlight the need for a surgery-specific classification system to address the clinical relevance and management of HO following PAO, as the existing classifications remain inadequate for this population.

The Brooker classification, originally developed for HO after THA,^16,17^ has been widely used but lacks specificity for the patterns observed following PAO. In our series, there were no patients categorized as grade 4, the most severe type in the Brooker classification system, but three of four patients requiring excision of heterotopic bone were classified as type 2. It has a limited ability to differentiate the severity and clinical impact of HO. The new classification system proposed in this study addresses these limitations by categorizing HO based on distinct radiological patterns specific to PAO. It achieved an AUC of 0.928, which indicated excellent predictive accuracy for symptomatic HO and was significantly higher than that of the Brooker classification (0.697). This approach not only enhances the granularity of classification, but also provides greater clinical relevance by associating specific subtypes with symptomatic presentations and the need for surgical intervention. The superior performance of the new classification, as demonstrated by ROC analysis, underscores its potential utility in clinical practice.

Adopting a classification system that better reflects the unique patterns of HO after PAO has important implications. It allows for improved risk stratification, enabling more targeted preoperative counselling and postoperative monitoring. Patients identified with higher-risk subtypes can be more closely monitored for symptoms or complications, potentially leading to earlier interventions and improved outcomes. Additionally, a tailored classification may facilitate more standardized reporting and comparison of outcomes across studies, advancing research in this area.

To date, there is no universally accepted standard regimen for preventing HO after hip surgery. Most research has focused on THA and hip arthroscopy. Both selective and non-selective NSAIDs have been reported to be effective for HO prevention, with similar efficacy between the two.^22-24^ One study indicated that the incidence of HO could be reduced to 1.5% when NSAIDs were used after hip arthroscopy with or without PAO.^10^ However, another study questioned the necessity of routine prophylaxis following hip arthroscopy due to the low incidence of HO.^25^ A meta-analysis further revealed that aspirin, commonly used for venous thromboembolism prophylaxis, also demonstrated effectiveness in preventing HO.^26^ Both regular-dose aspirin (325 mg per day) and low-dose regimens (81 mg or 162 mg per day) were found to be effective. In our cohort, where patients routinely took low-dose aspirin, additional NSAID prophylaxis demonstrated a trend towards reducing HO; however, the differences did not reach statistical significance. Notably, no patients in the prophylaxis group developed symptomatic HO or required excision, whereas a small number of cases occurred in the non-prophylaxis group. While these findings suggest a potential benefit of NSAID prophylaxis, the lack of statistical significance underscores the need for further prospective studies with larger sample sizes and controlled designs to establish definitive efficacy.

For HO causing significant symptoms, surgical excision is necessary and can be performed using either open or arthroscopic techniques. The choice of technique depends on the location, severity of the HO, and the surgeon’s expertise.^27^ We have only done HO resections in symptomatic patients with limited range of motion. Obtaining a bone scan demonstrating maturity of the HO is important prior to resection. While the procedure is straightforward, care must be taken to avoid irritating or injuring surrounding soft-tissues to minimize inflammation and reduce the risk of recurrence. Beyond NSAIDs, adjuvant radiotherapy can also be considered as a preventive measure to further lower the recurrence risk after excision.^28^

To address potential concerns regarding follow-up duration and selection bias, we conducted two sensitivity analyses. First, to evaluate the risk of missing early severe HO due to excluding patients without radiological follow-up within two months, we examined the timing of HO detection in the included cohort. Among 143 patients who developed HO, 48 cases (33.6%) were first identified on the two-month postoperative radiograph; all were classified as mild (types 1 or 2) and were asymptomatic. No cases of severe or symptomatic HO were detected at this early timepoint, suggesting that exclusion of these early follow-ups is unlikely to have missed clinically important HO. Second, we repeated the analysis excluding all patients with less than one year of follow-up (n = 181). In the remaining 462 patients, 109 (23.6%) developed HO, which is consistent with the full-cohort incidence (22.2%). These findings support the robustness of our results and indicate that the study’s conclusions are not significantly affected by follow-up duration or attrition.

This study had several limitations. Its retrospective design may have introduced bias, as data collection and analysis were reliant on pre-existing records. Additionally, patients underwent PAO with or without concomitant arthroscopic procedures, which were not standardized. However, we included these procedures as co-variables in the analysis and found no association between any specific arthroscopic intervention and the development of HO. The large scale of our cohort strengthens the credibility of the findings despite this variability. Our analysis was based on a single internal cohort, and the classification system has not yet undergone external validation. While the large sample size strengthens internal validity, future multicentre studies are needed to confirm its generalizability. Another limitation of our study was the absence of a standardized protocol for pharmaceutical prophylaxis for HO at our institution. NSAIDs were prescribed for prophylaxis in only a subset of patients, introducing the potential for selection bias. As a result, the relative effectiveness of prophylaxis should be interpreted with caution. This limitation reflects a broader gap in the literature, as no universally accepted protocol for HO prevention after PAO currently exists. Our newly proposed classification system provides a foundation for future studies to address this issue and develop standardized prevention strategies.

In conclusion, the cumulative probabilities of radiological and symptomatic HO at five years following PAO were 30.4% and 3.8%, respectively. Patients categorized as type 3 or type 4 in the proposed classification system demonstrated a 40% risk of developing symptoms and a 20% risk of requiring surgical excision. These findings support the concept that a tailored classification system enhances the understanding, assessment, and management of HO in the PAO population, providing a valuable framework for future research and clinical application.

**Take home message**

- This study establishes a new classification system for heterotopic ossification (HO) following periacetabular osteotomy, providing a more accurate correlation between radiological patterns and clinical symptoms than the Brooker system.

- The proposed system enables early identification of clinically significant HO (types 3 and 4), facilitating timely intervention and standardized reporting.

## Data Availability

The datasets generated and analyzed in the current study are not publicly available due to data protection regulations. Access to data is limited to the researchers who have obtained permission for data processing. Further inquiries can be made to the corresponding author.
